# Design, Analysis and Experiment of the Fiber Push-Out Device Based on Piezoelectric Actuator

**DOI:** 10.3390/mi12111420

**Published:** 2021-11-19

**Authors:** Mengxin Sun, Yong Feng, Jiangtao Xu, Xiaoyu Wang, Haojie Zhou

**Affiliations:** 1 Department of Mechanical Engineering, Nanjing Institute of Technology, Nanjing 211167, China; fengyong007@sina.cn (Y.F.); xutaowang007@163.com (J.X.); qq614064597@163.com (H.Z.); 2 College of Mechanical & Electrical Engineering, Nanjing University of Aeronautics and Astronautics, Nanjing 210016, China; wxy1962396971@163.com

**Keywords:** piezoelectric actuator, fiber push-out, in situ testing system design

## Abstract

In this study, a fiber push-out device based on a piezoelectric actuator was designed, analyzed and tested, and its experimental environment was designed. The piezoelectric actuator includes a flexible beam. By using response surface analysis (RSM), taking the large displacement as the objective function and on the premise of meeting the strength requirements, the structural parameters of the flexible beam were analyzed. In the process of fiber push-out, the interfacial shear stress was estimated by establishing the system integrating the fiber-matrix-composite three-phase model and the piezoelectric actuator model using the analytic method, and the theoretical analysis results of the fiber interface mechanical properties were given. A prototype of the system was made, and the performance tests of the piezoelectric actuator and the fiber push-out device were carried out. The test results showed that the designed piezoelectric actuator can achieve a stepping resolution of 6.67 μm and a maximum displacement of about 100 μm at the input voltage of 150 V, which is consistent with the design results. The extrusion test of a single fiber was carried out using a piezoelectric actuator. The mechanical properties of the interfacial layer during the push-out process were measured and the interfacial shear strength was calculated, which is consistent with the theoretical analysis results. Finally, based on the mechanical properties obtained from the test, the loading failure process of the fiber was simulated by finite element analysis, which well explained the failure process of the fiber, thus verifying the feasibility of the designed fiber push-out device.

## 1. Introduction

Fiber material has the advantages of a high specific strength, a high specific stiffness and a high specific modulus, which can be used in the production of blades, casing and other aerospace components for its capacity of effectively reducing the weight of the aero-engine and improving the working efficiency of the aircraft [1]. In addition, some fiber materials are used in the field of energy harvest and human health monitoring. For instance, piezoelectric fiber from a melt spun process with the ability of mass production and flexibility are used in triaxial braided piezo fiber energy harvesters developed by Fatemeh Mokhtari et al. [2,3,4] The interface layer between fiber and matrix is formed in the process of material preparation, and its bonding properties often directly affect the overall mechanical properties of composites. Studying the mechanical properties of the interface can reveal the influence of interface structure, properties and stress on mechanical properties, which provides a basis for further understanding the deformation and failure mechanism of composite materials [5]. At present, the commonly used interface testing methods include fiber pull-out [6,7], droplet debonding [8,9,10], fiber breaking [11] and fiber push-out [12,13,14]. Among them, the fiber push-out test, as a method for in-situ test of real materials with a need to prepare special specimens, can conveniently detect the change of interface properties through a load displacement curve. This method is favored by the majority of researchers and has been widely studied in the past decade. Marshell, for the first time, used the spherical probe and Vickers microhardness probe to apply a load on a single fiber, and estimated the interfacial shear strength of the composite material [15]. By thinning the sample thickness, the fiber was completely extruded eventually. W.M.Mueller conducted push-out tests on the composites with different indenters based on nano-indentation instruments to determine the interfacial shear strength [16]. Magali Rollin et al. carried out push-out tests on the specimens with certain inclination angles using a diamond indenter, and obtained the interfacial shear strength of the composites according to the load-displacement curves [17]. From the above investigations, it can be seen that the fiber push-out experiment belongs to the in-situ test method of composite materials. The main equipment used in the traditional push-out experiment is a nano indentation instrument or a hardness tester with diamond or other hardness probes. With the decrease in fiber diameter and the increase in interfacial shear strength, the indenter volume and precision of the original instrument may be affected, and there may be fiber breakage and the probe may not be able to always contact the fiber during the push-out process. Therefore, it is difficult to properly push out the whole fiber on the sheet and obtain high-precision experimental data of fiber extrusion, thus failing to guide the judgment of failure mode.

A piezoelectric actuator has been widely used in many fields due to the advantage of high precision, fast response and a large thrust-to-weight ratio. In the field of in-situ testing, piezoelectric devices are often used as high-precision testing devices to collect relevant data in material testing [18]. Some piezoelectric devices are also used to replace the probes of the original equipment. U. Rabe et al. developed a nano-indentation tester integrating a piezoelectric actuator, in which large strokes are actuated by a stepper motor and a precise feed motion is achieved by a piezoelectric actuator [19]. Wang Shupeng et al. developed a micro device based on bionic piezoelectric actuators, with which the static tensile test and dynamic fatigue test is carried out respectively. Then the results are compared with the test data from commercial tensile testers to verify the feasibility of the device [20]. Huang Hu et al. designed a piezoelectric flexible hinge drive unit that can be used as a probe for self-made indentation equipment [21]. Ma Zhichao et al. focused on a thermos-mechanical coupled in situ fatigue device driven by a piezoelectric actuator, of which the structural resonances, transient response, grip design and thermal insulation performance are discussed in detail [22]. Moreover, the fatigue life test is carried out, and the feasibility of the device is verified. From the above researches, it can be seen that researches on the design of piezoelectric push-out devices are only limited to the manufacturing of the devices, and the judgment on the accuracy of the results, and the analysis of the material failure is lacking. Compared with the mature traditional test methods, the accuracy still needs further analysis and verification.

In this paper, a fiber push-out device based on piezoelectric actuators is proposed, and the structure is optimized with the aim of a large stroke. The prototype was designed and machined, and the SIC long fiber reinforced a titanium matrix composite, of which the interfacial strength can hardly be analyzed by the traditional in-situ test method, was selected for experimental analysis. The experimental results were compared with the pre-analysis results. At the same time, the failure mechanism and process of fiber composite material were simulated based on the results obtained, which further confirms the feasibility of the proposed fiber extrusion device. Compared with the existing fiber push-out device, the proposed piezoelectric driven ejector can meet the performance test requirements of finer and thinner fiber composites, and is conducive to more accurate calibration of composite material parameters.

## 2. Structure and Operational Principle

### 2.1. Structural Design of Actuator

**Figure 1:**
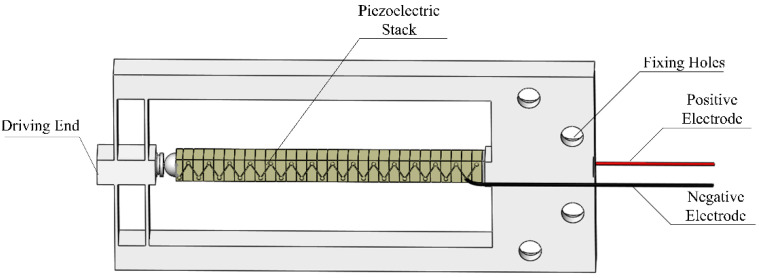
Structure of the piezoelectric actuator.

Considering the thickness of the fiber glass and the diameter of the single fiber, a proper piezoelectric actuator for loading is the key to the design. In the design of direct-driven piezoelectric actuators for loading, stiffness and displacement are two important factors that restrict each other, and significantly affect the accuracy, stroke and response speed of the piezoelectric actuator during the push-out process. As shown in Figure 1, for the piezoelectric actuator structure used, the laminated piezoelectric ceramics are pressed in the frame structure, and the positive and negative wires are respectively drawn from the two sides. One side of the frame structure is equipped with two sets of driving ends connected by parallel beams, and the other side is arranged with a through hole for fixing.

### 2.2. Design of the Push-Out Device

**Figure 2:**
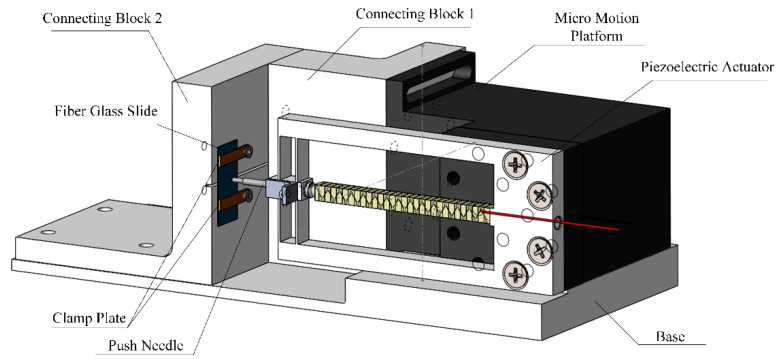
Structure of the push-out system.

In order to achieve the desired effect of fiber extrusion, the structure of the whole device was designed considering the structure of the piezoelectric actuator. As shown in Figure 2, the main structure of the device consists of a piezoelectric actuator, a pre-adjusted micro-actuator platform, L-shaped connecting blocks 1 and 2, and a base. The piezoelectric actuator is connected to the manual adjustable micro-displacement platform through screws. The manual adjustable micro-motion platform and L-shaped connecting block 2 are fixed on the base, and the side ends are fixed on one side of the L-shaped connecting block 1. L-shaped connecting block 1 is fixedly connected to the working face of L-shaped connecting block 2. The working surface of L-shaped connecting block 2 is arranged with fiber glass slides fastened by pressing plates and pressing screws, and a kert is arranged in the middle to store the extruded fibers. The size of the fiber glass sheet used is 36 × 17 × 0.3 mm.

**Figure 3:**
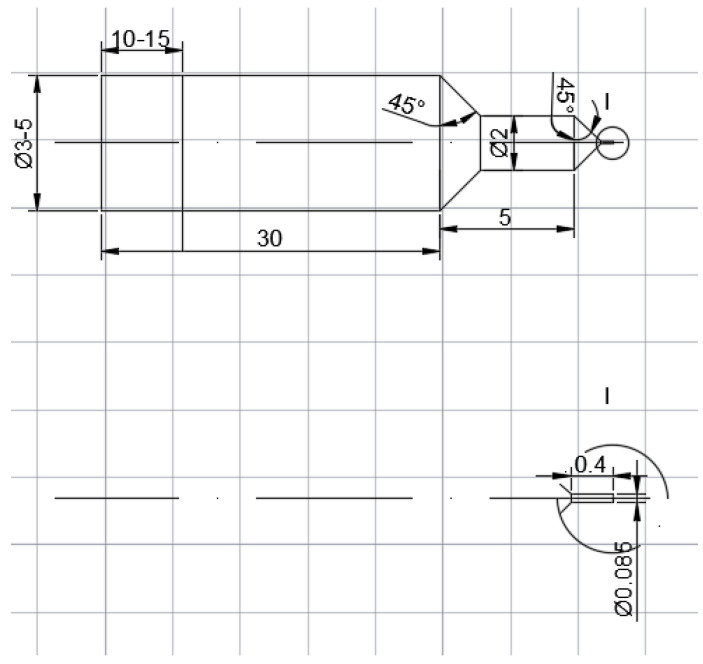
Dimension of the ejector pin.

In order to successfully push out the fiber, a suitable ejector pin should be arranged in the front end of the piezoelectric actuator. Since the fiber in the material used is about 100 μm in diameter, the ejector pin of ordinary materials cannot meet such a small scale. Moreover, the ejector pin needs to have a strong stiffness, so that an ejector pin modified from a small tungsten steel milling cutter is adopted, with the dimension shown in Figure 3.

The pre-adjusting micro-motion platform is manually controlled, placed on the base, and the side ends are fixedly connected to the L-shaped connecting block 1. The function of the pre-adjusted micro-platform is to control the ejector pin to approach the fiberglass slowly before the push-out experiment begins. The experiment was carried out under microscopical observation until the ejector pin was aligned with a single fiber.

**Figure 4:**
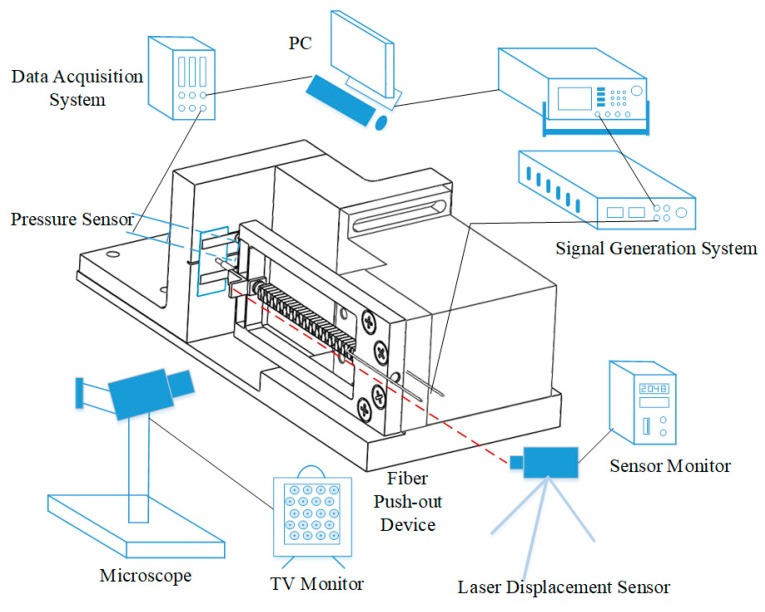
Test system.

The test system design is shown in Figure 4. The signal generator and power amplifier were used to provide the electrical signals required for the normal output of piezoelectric ceramics. A laser displacement sensor was used to measure the real-time displacement of the extrusion mechanism. A pressure sensor was placed at the lower part of the fiberglass to measure the real-time compressive stress data. A microscope was placed to observe the contact between the ejector pin and the fiberglass, checking their alignment and push-out conditions.

## 3. Theoretical Analysis

### 3.1. Optimization of the Piezoelectric Actuator

**Figure 5:**
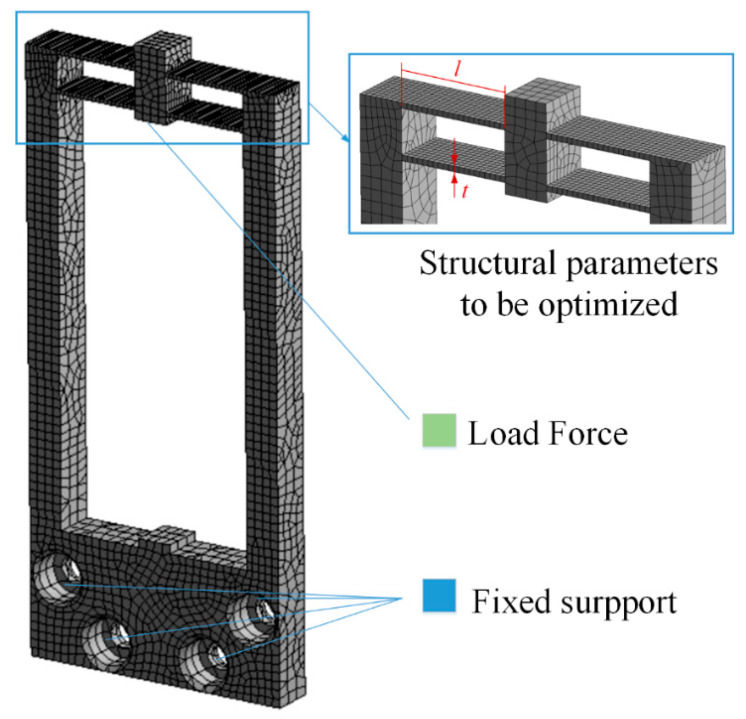
Mesh and load of the model.

The output structure of the piezoelectric actuator is optimized by using the finite element method, and the meshing results are shown in Figure 5. The hex dominant method is used in meshing when the body size is defined as 1 mm, and the element number is 186437. In FEM models, the four mounting holes are set as fixed connections, and forces are loaded in two elongation directions of piezoelectric ceramics. Since the piezoelectric mechanism is operated at a low frequency, static analysis is picked to obtain the results.

**Figure 6:**
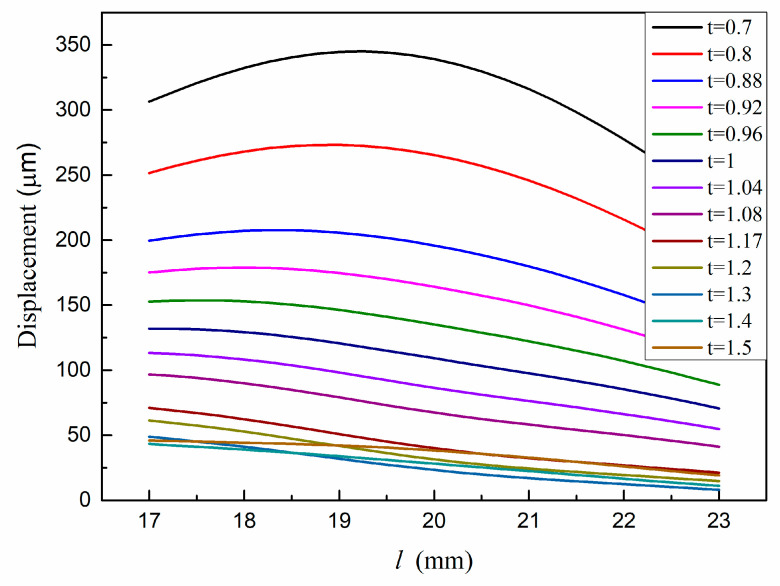
Displacement of the driving end versus structural parameters.

**Figure 7:**
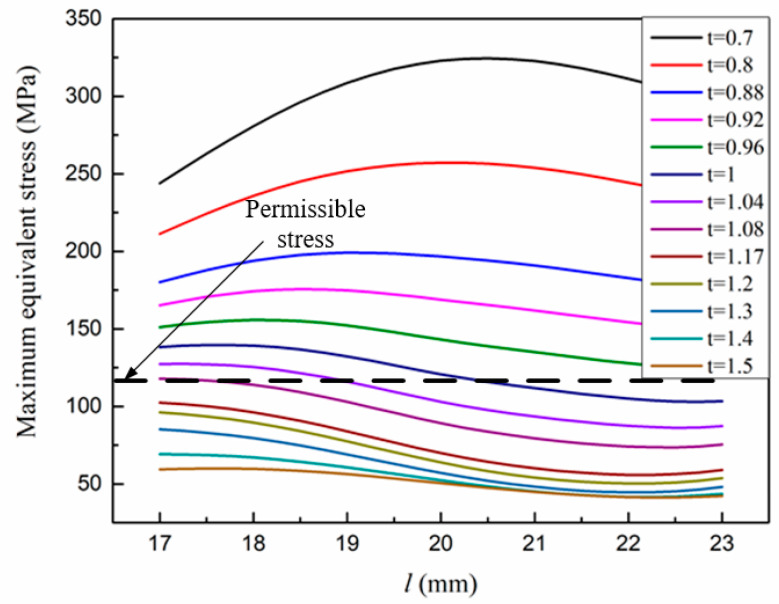
Maximum equivalent stress structural parameters versus.

The displacement of the output end and the maximum stress of the structure are taken as the objective functions, and the optimization parameters are the thickness and length of the parallel beams in Figure 5. The analysis results are shown in Figure 6 and Figure 7. It can be seen that when the thickness increases, both displacement and maximum stress decrease; when the length increases, both displacement and stress increase. In the allowable range of stress, the thickness of the parallel beam is 1 mm and the length is 20.5 mm.

**Figure 8:**
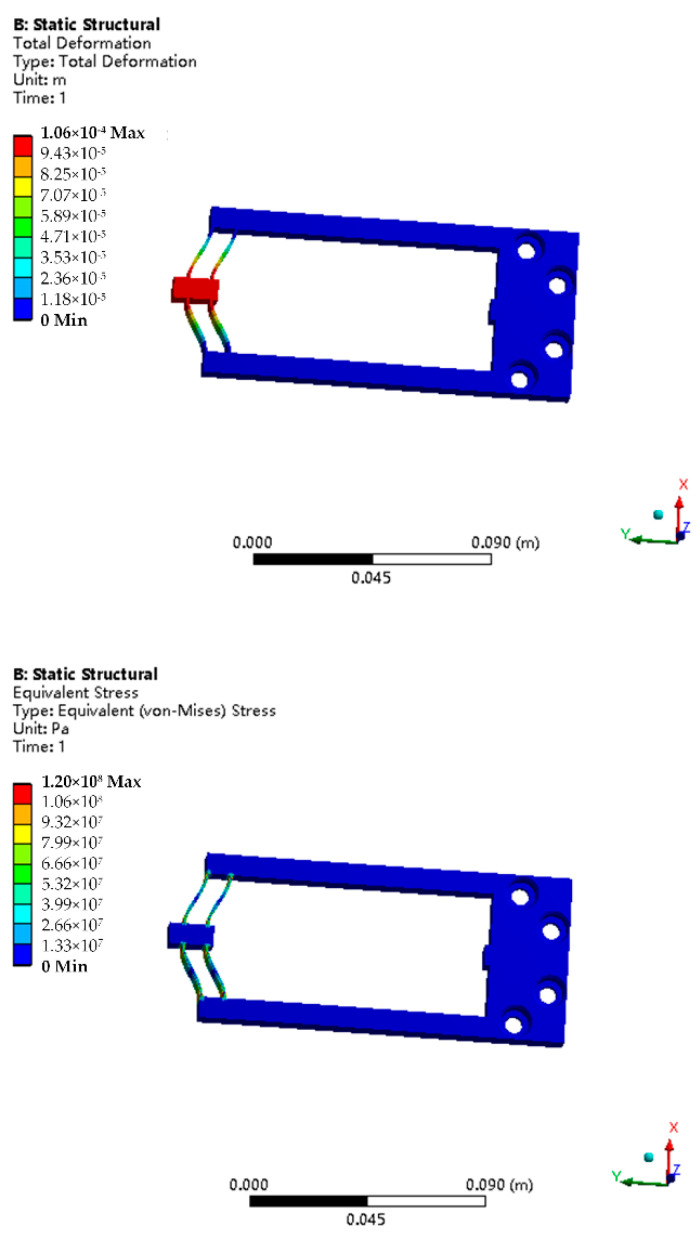
Maximum deformation and equivalent stress of the structure.

Under the selected size parameters, the input/output validation of the structure was carried out, and the results are shown in Figure 8.

**Figure 9:**
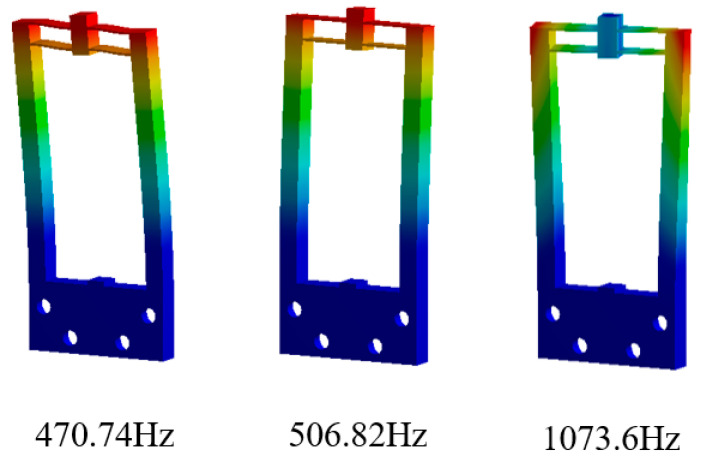
The first three modes of the structure.

The significance of modal analysis lies in avoiding the nonlinear change of structure due to resonance and reducing the difficulty in signal control. The finite element method was used for modal analysis. As shown in Figure 9, the first three modes can be obtained, which are the lateral bending vibration with the first frequency of 470.74 Hz, the normal bending vibration with the second frequency of 506.82 Hz and the torsional vibration with the third frequency of 1073.6 Hz. Since the first mode frequency of the frame structure is 470.74 Hz, the structure can operate stably at lower frequencies.

Table 1 with the main materials and dimensions of the piezoelectric mechanism is attached.

### 3.2. Analysis of Interfacial Shear Stress

**Figure 10:**
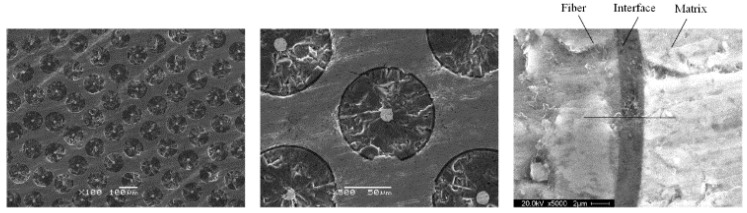
Micro-morphology of SIC long fiber titanium matrix reinforced composite material.

The micro-morphology of SIC long fiber titanium matrix-reinforced composite material used in the experiment is shown in Figure 10. This fiber composite was conducted by vacuum hot pressing or hot isostatic pressing after twining titanium wire and fiber. The fiber, central tungsten rod, interfacial layer and matrix can all be observed in the figure as well.

**Figure 11:**
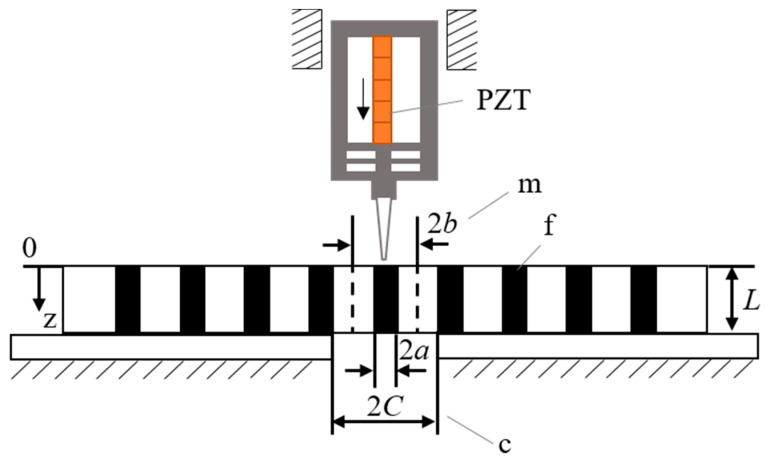
Theoretical model of the system.

The interfacial strength of the fiber push-out process can be described and predicted by a fiber-matrix-composite three-phase model. As shown in Figure 11, the fiber glass slide sample is placed on a slotted base and a three-cylinder model is adopted.

The load is applied to the fiber by a piezoelectric push-out device. At the supporting end of the sample, the fiber remains free, and the supporting platform supports the surrounding composite. The analytical model is a three-cylinder model. Where, *a*, *b*, *C* is the radius of the fiber in the center of the cylinder, the radius of the middle matrix and the radius of the outermost elastic composite, *V*_f_ is the volume fraction of fiber, *L* is the thickness of sample. The cylindrical coordinate system (*r*, *θ*, *z*) is adopted. *ε*, *σ* and *τ* are defined as the strain, normal stress and shear stress, and the subscripts f, m and c are fiber, matrix and composites, respectively. The volume ratio of fiber to matrix and the volume ratio of matrix to composite are,

(1) $$\gamma =a^{2}/(b^{2}-a^{2}),$$

(2) $$\gamma_{1}=b^{2}/(C^{2}-b^{2}),$$

The pressure *P* applied on a single fiber comes from the laminated piezoelectric ceramic actuator. If the applied voltage is *U*(*t*), the pressure can be expressed as:

(3) $$P=nd_{33}U(t)k_{P},$$

where, *n* is the number of layers, *d*_33_ is the piezoelectric constant and *k*_P_ is the stiffness of the piezoelectric ceramic. If the sectional area of fiber is *A* = *πa*^2^, when the compressive stress *σ* = *P*/*A* is applied axially, the axial stress on the fiber is transferred from the fiber to the transversely isotropic composite of the outer matrix and the outermost layer through the interfacial shear stress between the fiber and the matrix, and between the matrix and the composite. The axial stress of fiber, matrix and composite is assumed as $\sigma_{f}^{z}$, $\sigma_{m}^{z}$, $\sigma_{c}^{z}$, the equilibrium equation in the axial direction of the fiber can be obtained.

(4) $$\sigma_{f}^{z}(z)+\frac{1}{\gamma }\sigma_{m}^{z}(z)+\frac{1}{V_{f}\gamma_{1}}\sigma_{c}^{z}(z)=\sigma_{P}\sigma_{P}=\frac{P}{\pi a^{2}},$$

The ratio of Young’s modulus of matrix to fiber, the ratio of the Young’s modulus of the matrix to that of the composite, the ratio of Young’s modulus of matrix to composite and the Ratio of Young’s modulus of matrix to fiber are defined as,

(5) $$\alpha =E_{m}/E_{f},$$

(6) $$\alpha_{1}=E_{m}/E_{c}^{r},$$

(7) $$\alpha_{2}=E_{m}/E_{c},$$

(8) $$\alpha_{3}=E_{m}/E_{f}^{r},$$

The Poisson’s ratio is *υ*. Based on the relationship between the axial stress and the shear stress, the radial stress at the fiber–matrix interface and the matrix–composite interface can be obtained.

(9) $$q_{a}(z)=\frac{1}{V_{f}k_{3}}\left\{V_{f}k_{2}\alpha \upsilon_{f}\sigma_{f}^{z}(z)-[V_{f}k_{2}\upsilon_{m}-2\gamma \upsilon_{m}+2\gamma \alpha_{1}\upsilon_{c}]\sigma_{m}^{z}(z)\right\},$$

(10) $$q_{b}(z)=\frac{1}{k_{3}}[2\gamma \alpha \upsilon_{f}\sigma_{f}^{z}(z)-k_{4}\sigma_{m}^{z}(z)],$$

where,

(11) $$k_{1}=\frac{\alpha \upsilon_{f}+\gamma \upsilon_{m}}{\alpha_{3}(1-\upsilon_{{}_{f}}^{r})+1+2\gamma +\upsilon_{m}},$$

(12) $$k_{2}=1+2\gamma -\upsilon_{m}+\alpha_{1}(1+2\gamma_{1}+\upsilon_{{}_{c}}^{r}),$$

(13) $$k_{3}=\frac{1}{V_{f}}\left\{V_{f}k_{2}[\alpha_{3}(1-\upsilon_{{}_{f}}^{r})+1+2\gamma +\upsilon_{m}-4\gamma^{2}\right\},$$

(14) $$k_{4}=2\gamma \upsilon_{m}-(\upsilon_{m}-\alpha_{2}\upsilon_{c})[\alpha_{3}(1-\upsilon_{f}^{r}+1+2\gamma +\upsilon_{m}],$$

If the interface crack propagates from the support end (*z* = *L*) to the loading end (*z* = 0) and the length of the crack is l when the interface is completely debonded, the fiber can be divided into three regions according to the length direction: debonding region 1 (*L*-*l* ≤ *z* ≤ *L*), crack tip region 2 (*L*_1_ ≤ *z* ≤ *L*-*l*) and bonding region 3 (0 ≤ *z* ≤ *L*_1_). Region 2 has a small length and no axial stress distribution. The axial stresses of the other two regions have been described in the other literatures.

The axial stress on the fiber in the debonding area is as follows.

(15) $$\sigma_{f}^{z}(z)=\frac{2\tau_{\mathrm{fr}}(L-z)}{a},$$

The axial stress on the fiber in the bonding area is,

(16) $$\begin{array}{l} \sigma_{f}^{z}(z)=\frac{1}{\sinh(\sqrt{A_{1}}(L-l)}[(\frac{A_{2}}{A_{1}}\sigma_{p}+\sigma_{f(L-l)})\sinh(\sqrt{A_{1}}z)\\ -(1+\frac{A_{2}}{A_{1}})\sigma_{P}\sinh(\sqrt{A_{1}}(z+l-L))]-\frac{A_{2}}{A_{1}}\sigma_{P} \end{array}$$

where,

(17) $$\begin{array}{ll} A_{1}= & \frac{2\gamma }{V_{f}k_{3}\gamma_{2}(1+\upsilon_{m})[2\gamma C^{2}\ln(b/a)-a^{2}]}\\ & \left\{\begin{array}{l} {}[V_{f}k_{3}(\alpha +\gamma_{2})-2(\alpha \upsilon_{f}+\gamma \upsilon_{m})[V_{f}k_{2}\alpha \upsilon_{f}\\ +\gamma_{2}(V_{f}k_{2}\upsilon_{m}-2\gamma \upsilon_{m}+2\alpha_{1}\gamma \upsilon_{c})]+V_{f}\upsilon_{m}(2\gamma \alpha \upsilon_{f}+\gamma_{2}k_{4})] \end{array}\right\} \end{array}$$

(18) $$\begin{array}{l} A_{2}=\frac{-2\gamma }{V_{f}k_{3}(1+\upsilon_{m})[2\gamma C^{2}\ln(b/a)-a^{2}]}\\ \left[V_{f}k_{3}-2(\alpha \upsilon_{f}+\gamma \upsilon_{m})(V_{f}k_{2}\upsilon_{m}-2\gamma \upsilon_{m}+2\alpha_{1}\gamma \upsilon_{c})+V_{f}k_{4}\upsilon_{m}\right] \end{array}$$

The relationship between the axial stress of fiber–matrix and its interfacial shear force is shown as follows.

(19) $$\frac{d\sigma_{f}^{z}(z)}{dz}=-\frac{2}{a}\tau_{a}(z),$$

where, *τ_a_*(*z*) is the interfacial shear stress between fiber and matrix.

Finally, the interfacial shear stress caused by load *P* in region 3 can be obtained as,

(20) $$\begin{array}{l} \tau_{\mathrm{III}}^{P}(z)=-\frac{a\sqrt{A_{1}}}{2\sinh(\sqrt{A_{1}}(L-l))}[(\sigma_{L-l}+\frac{A_{2}}{A_{1}}\sigma_{P})\\ \cosh(\sqrt{A_{1}}z)-(1+\frac{A_{2}}{A_{1}})\sigma_{P}\cosh(\sqrt{A_{1}}(z+l-L))] \end{array}$$

The reference values of the selected parameters of the analytical model are shown in the Table 2.

**Figure 12:**
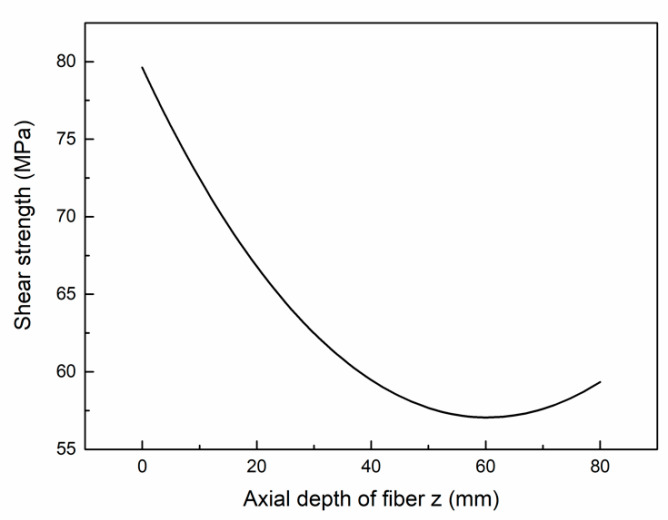
Interfacial shear stress versus axial direction in the bonding region.

The curves of the interfacial shear stress versus axial direction in the bonding region are shown in the Figure 12. According to the theoretical results, the interfacial shear strength is 79.62 MPa.

## 4. Experiment 

### 4.1. Experiment Environment

**Figure 13:**
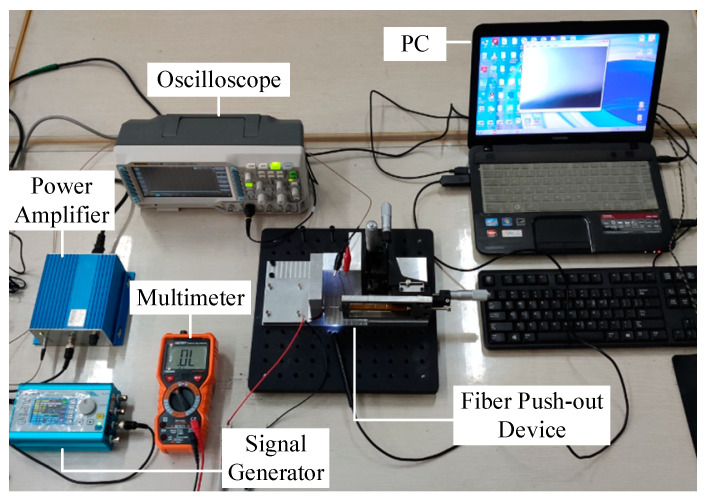
Experiment environment.

The experiment environment is shown in the Figure 13.

**Figure 14:**
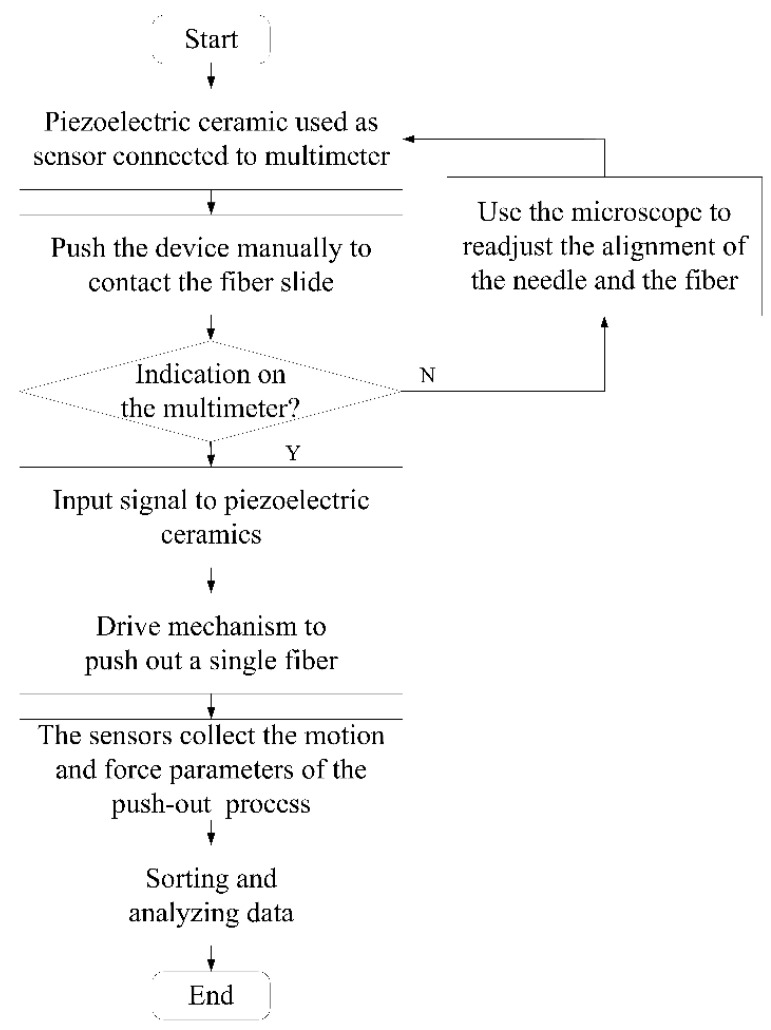
Test flow chart.

At the beginning of the formal test, the positive and negative electrodes of the laminated piezoelectric ceramics were connected to the multimeter, and the micro platform was adjusted to make the ejector pin approach the fiber glass sheet slowly. The alignment of the ejector pin and the fiberglass was observed through a microscope, and the micro platform was continuously adjusted until the multimeter showed numbers. The driving signal of the linearly increasing voltage was input into the laminated piezoelectric ceramics through a power amplifier, and the piezoelectric actuator was driven to extrude the pin needle forward until the single fiber extruded the glass sheet. At the same time, the displacement changes at the top of the micro displacement output unit and the output changes of the thin film pressure sensor were collected, and the relevant parameters of the fiber extrusion process were calculated according to the output characteristics of the pressure sensor. The specific testing process is shown in Figure 14.

### 4.2. Test of the Piezoelectric Actuator

**Figure 15:**
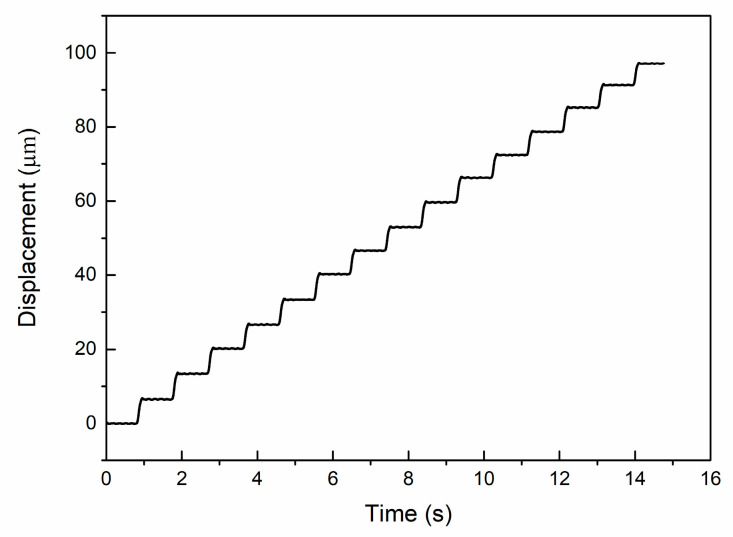
The performance of the piezoelectric actuator.

The performance of the piezoelectric actuator directly affects the test quality of the extrusion process, so the performance test of the piezoelectric actuator was carried out first. The step wave driving signal as shown in the figure above was input into the laminated piezoelectric ceramics, and the displacement output of the piezoelectric actuator was measured by the laser displacement sensor at the output end, so that the displacement–time curve can be obtained, as shown in Figure 15 below. Combined with the input signals, it can be seen from the figure that when the voltage of 150 V is applied to the laminated piezoelectric ceramics, the actuator motion is about 100 μm and the stepping displacement is about 6.67 μm. The voltage and displacement of piezoelectric actuator showed a high degree of linearity.

### 4.3. Fiber Push-Out Test

**Figure 16:**
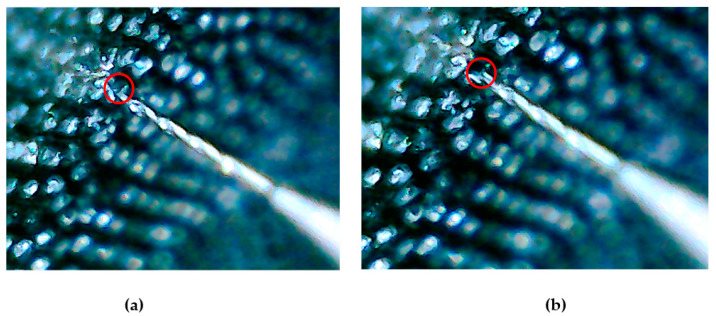
(**a**) The micro morphology before push-out test; (**b**) The micro morphology after push-out test.

The part of the ejector pin and the fiberglass sheet under the microscope before and after extrusion is shown in Figure 15. As shown in Figure 16a, each bright spot contains a fiber. The ejector pin at the front of the device is aligned with one of the fibers. The alignment process mainly depends on a microscope and a manual fine-tuning device. As can be seen from Figure 16b, the ejector pin driven by the piezoelectric actuator has completely pushed the single fiber out, forming a new gap on the fiber glass.

**Figure 17:**
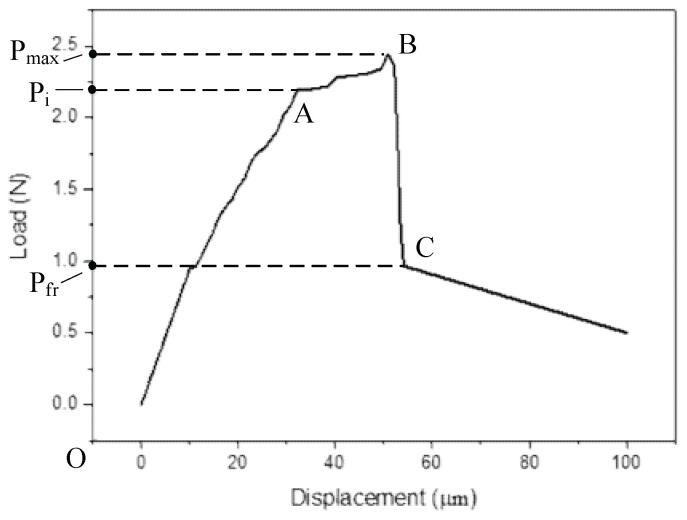
The force applied on the ejector pin versus its motion displacement in the push-out process.

According to the data collected by the pressure sensor and combining the motion curve of the piezoelectric actuator, the relationship between the force applied on the ejector pin and its motion displacement in the push-out process can be obtained, as shown in Figure 17.

The accuracy of the test method is verified by analyzing the results in Figure 16 above, and the results in Figure 16 are discussed in the next section.

### 4.4. Results Analysis

In the diagram of the push-out test, the abscissa is the fiber displacement and the ordinate is the measured applied load. According to the previous studies, the load at A represents the initial debonding load, the load at B represents the maximum load, and the load at C is the load applied only to overcome the sliding friction stress after the section is completely debonded. The fiber movement process can be divided into several stages.

In the process from O to A, the applied load increases, the interface shear stress reaches the critical value, and the interface begins to debond. In the process from A to B, after debonding occurs, with the increase of the applied load, the interface shear stress decreases and the maximum value begins to transfer, and further debonding of the interface occurs.

In the process from B to C, when the load reaches the maximum, the maximum shear stress reaches the critical value at the bottom of the glass sheet, the interface is completely debonded, and the fiber is extruded from the matrix.

According to the shear stress formula,

(21) $$\tau =\frac{P}{\pi d_{f}L},$$

by substituting the maximum load in the experiment and the stress load after debonding into the formula, the interfacial shear strength and the average friction stress can be calculated to be 76.43 MPa and 31.85 MPa respectively, which is basically consistent with the analytical results of the model.

**Figure 18:**
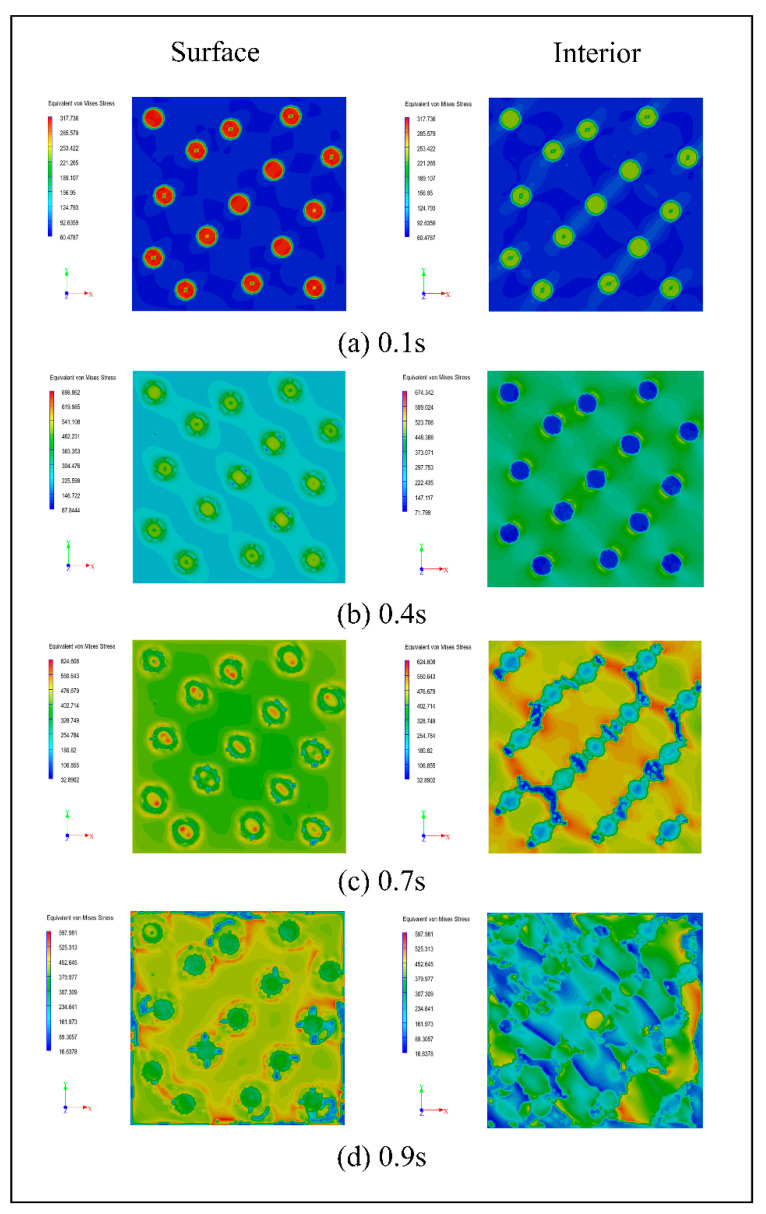
The stress change process of the composite material at (**a**) 0.1 s; (**b**) 0.4 s; (**c**) 0.7 s; (**d**) 0.9 s.

By using interfacial shear strength and other material parameters, the failure modes of materials can be roughly judged and analyzed. By establishing the fiber model using a finite element method, introducing the shear stress intensity parameters measured by the piezoelectric fiber push-out device, and increasing stress on the material surface within 1 s, the stress change process can be obtained, as shown in Figure 18.

The stress nephogram shows that the initial stress is small, and that the stress is basically concentrated on the fiber, and the surface and the matrix of the material only bears a small force. With the increase in the external force, the shear strength of the interface layer between the fiber and the matrix soon reaches the limit at 0.4 s, and the surface matrix begins to bear the external force, while the force of the fiber inside the material decreases. When the external force continues to increase, the force on the surface fiber is slightly higher than that on the matrix at 0.7 s, the internal fiber buckles, and only small cracks appear on the matrix close to the fiber. After continuous loading, the stress on the surface matrix exceeds the strength of the fiber, and the internal fiber breaks and fails completely, resulting in cracks in propagation on the matrix.

The simulation results show that the lower interfacial bonding strength can delay the crack propagation when the fiber composite is longitudinally loaded. The simulation analysis based on push-out test parameters can well explain the loading failure process of fiber composites.

## 5. Conclusions

In this paper, a fiber push-out device based on piezoelectric actuators is designed, analyzed and tested. The designed fiber push-out device needs to push out fibers with a diameter of only 100 μm, and the average thickness of fiber glass is only 300 μm. It is difficult to meet this test requirement by using the traditional in-situ tester. Therefore, a piezoelectric driver with a higher precision is used to measure the pushing force in the process of completely pushing out the fiber by using the micro step motion signal. Taking large displacement and sufficient strength as the objective function, the main flexible hinge parameters in the piezoelectric actuator are optimized by using the finite element method, and the shear strength of the fiber in the bonding stage is predicted by using the fiber-matrix-composite three-phase model and the basic piezoelectric equation. At the same time, the overall design of the experimental environment of the test device is carried out, and the output performance of the piezoelectric actuator during the single fiber push-out process is tested. According to the theory and simulation, the interface mechanical properties of the fiber push-out process are analyzed, and the failure process of the fiber composite is further explained by simulation based on the obtained performance parameters, which verifies the accuracy of the piezoelectric actuator-based fiber extrusion device. The conclusions of this study are summarized as follows:

(1) In the optimization design of the piezoelectric actuator, the displacement of the output end and the maximum stress of the structure are taken as objective functions, and the thickness and length of the parallel beams are set as independent variables. According to the optimization results of finite element software, when the thickness of the parallel beam is 1 mm and the length is 20.5 mm, the piezoelectric micro-displacement actuator can be operated stably and accurately under a low frequency (less than 150 Hz).
(2) Based on the three-phase, three-cylinder model of fiber-matrix composite and the basic equation of piezoelectric actuator, the interfacial shear stress formula between fiber and matrix is obtained. The shear stress-axial length curve of the bond zone is obtained by substituting the parameters into the formula, and the theoretical interfacial shear strength is 79.62 MPa.
(3) The flow of fiber push-out test is developed. The performance test of the designed piezoelectric actuator shows that the maximum displacement of the actuator is about 100 μm and the stepping displacement is about 6.67 μm when the voltage signal of 150 V is input. There is a good linearity between the input voltage and the output displacement. The fiber extrusion process can be divided into three stages. Through analysis, the interfacial shear strength is 76.43 MPa, and the average friction stress is 31.85 MPa, which is basically consistent with the theoretical analysis results.
(4) Based on the test results, further finite element tests are carried out to analyze the failure mechanism of SIC long fiber reinforced titanium matrix composites during longitudinal loading. It is concluded that the lower interfacial bonding strength can delay the crack propagation, and the results of piezoelectric actuator-based fiber push-out test provide a data basis for summarizing the properties of composite materials.

## Figures and Tables

**Table 1:** Main materials and dimensions of the piezoelectric mechanism.

Parameters	Values	
	Metal Elastic Mechanism (Stainless Steel)	Piezoelectric Ceramics
Density (kg/m^3^)	7900	7640
Elasticity modulus (Pa)	2 × 10^11^	-
Poisson’s ratio	0.3	0.31
*d*_33_ (m/V)	-	7.2 × 10^−10^
Size	140 mm × 64 mm × 10 mm (overall size)	7 mm × 7 mm × 93 mm
	100 mm × 48 mm × 10 mm (center frame size)	-
	20.5 mm × 1 mm × 10 mm (Cantilever beam size)	-

**Table 2:** Reference values of the selected parameters of the analytical model.

*a* (μm)	*b* (μm)	*C* (μm)	*L* (μm)	*l* (μm)
50	100	200	100	20
*E*_f_ (GPa)	$E_{f}^{r}$ (GPa)	*E*_m_ (GPa)	*E*_C_ (GPa)	$E_{C}^{r}$ (GPa)
469	400	110	325.4	284
*υ*_f_	$\upsilon_{f}^{r}$	*υ*_m_	*υ*_C_	$\upsilon_{C}^{r}$
0.17	0.16	0.26	0.206	0.2
*d*_33_ (×10^−12^ m/V)	*U*max (V)	*k*_P_ (×10^6^ N/m)	*μ*	
720	150	6	0.3	

## Nomenclature

*l*	The length of the parallel beam
*t*	The length thickness of the parallel beam
*a*	The radius of the fiber in the center of the cylinder, *t*
*b*	The radius of the middle matrix
*C*	The radius of the outermost elastic composite
*γ*	The volume ratio of fiber to matrix
*γ*_1_	The volume ratio of matrix to composite
*P*	Pressure
*n*	The number of layers
*d*_33_	The piezoelectric constant
*U(t)*	Applied voltage
*k_p_*	The stiffness of the piezoelectric ceramic
$\sigma_{f}^{z}$	The axial stress of fiber
$\sigma_{m}^{z}$	The axial stress of matrix
$\sigma_{c}^{z}$	The axial stress of composite
*V*_f_	The volume fraction of fiber
*L*	The thickness of sample
*σ_P_*	The compressive stress
*E*_f_	The young’s modulus of fiber
*E*_m_	The young’s modulus of matrix
*E*_C_	The young’s modulus of composite
*υ*_f_	The Poisson’s ratio of fiber
*υ*_m_	The Poisson’s ratio of matrix
*υ*_C_	The Poisson’s ratio of composite
μ	Friction coefficient
